# Divergent gene expression responses in two Baltic Sea heterotrophic model bacteria to dinoflagellate dissolved organic matter

**DOI:** 10.1371/journal.pone.0243406

**Published:** 2022-11-17

**Authors:** Christofer M. G. Osbeck, Daniel Lundin, Camilla Karlsson, Jonna E. Teikari, Mary Ann Moran, Jarone Pinhassi

**Affiliations:** 1 Centre for Ecology and Evolution in Microbial Model Systems, EEMiS, Linnaeus University, Kalmar, Sweden; 2 Department of Microbiology, University of Helsinki, Helsinki, Finland; 3 Department of Agricultural Sciences, University of Helsinki, Helsinki, Finland; 4 Department of Marine Sciences, University of Georgia, Athens, Georgia, United States of America; University of Utah, UNITED STATES

## Abstract

Phytoplankton release massive amounts of dissolved organic matter (DOM) into the water column during recurring blooms in coastal waters and inland seas. The released DOM encompasses a complex mixture of both known and unknown compounds, and is a rich nutrient source for heterotrophic bacteria. The metabolic activity of bacteria during and after phytoplankton blooms can hence be expected to reflect the characteristics of the released DOM. We therefore investigated if bacterioplankton could be used as “living sensors” of phytoplankton DOM quantity and/or quality, by applying gene expression analyses to identify bacterial metabolisms induced by DOM. We used transcriptional analysis of two Baltic Sea bacterial isolates (*Polaribacter* sp. BAL334 [*Flavobacteriia*] and *Brevundimonas* sp. BAL450 [*Alphaproteobacteria*]) growing with DOM from axenic cultures of the dinoflagellate *Prorocentrum minimum*. We observed pronounced differences between the two bacteria both in growth and the expressed metabolic pathways in cultures exposed to dinoflagellate DOM compared with controls. Differences in metabolic responses between the two isolates were caused both by differences in gene repertoire between them (e.g. in the SEED categories for membrane transport, motility and photoheterotrophy) and the regulation of expression (e.g. fatty acid metabolism), emphasizing the importance of separating the responses of different taxa in analyses of community sequence data. Similarities between the bacteria included substantially increased expression of genes for Ton and Tol transport systems in both isolates, which are commonly associated with uptake of complex organic molecules. *Polaribacter* sp. BAL334 showed stronger metabolic responses to DOM harvested from exponential than stationary phase dinoflagellates (128 compared to 26 differentially expressed genes), whereas *Brevundimonas* sp. BAL450 responded more to the DOM from stationary than exponential phase dinoflagellates (33 compared to 6 differentially expressed genes). These findings suggest that shifts in bacterial metabolisms during different phases of phytoplankton blooms can be detected in individual bacterial species and can provide insights into their involvement in DOM transformations.

## Introduction

Dissolved organic matter (DOM) in seawater is estimated to represent one of the largest reservoirs of organic carbon on earth [1]. It consists of a complex mixture of compounds of different molecular weights, solubility and volatility [2] and is traditionally classified according to bioavailability (i.e. labile, semi-labile and refractory) with turnover times ranging from minutes to thousands of years [3]. DOM released by living and dying phytoplankton is an important source of organic carbon available for heterotrophic bacteria [4], and it was recently shown that exudates as compared to lysates from phytoplankton is preferentially used by distinct components of marine bacterioplankton [5]. Up to 50% of the carbon fixed by primary producers in marine and limnic ecosystems–bacterial and eukaryotic phytoplankton as well as multicellular algae–is turned over by bacterioplankton in the microbial loop [6]. This way, organic carbon is degraded and transformed by the microbial community, with most eventually respired as CO_2_. This carbon turnover occurs at a rate that is orders of magnitude higher in the sea than in terrestrial ecosystems [7, 8], particularly so in coastal environments and inland seas where nutrient concentrations do not limit microbial activities to the same extent as in the open ocean. Given the tight linkages between phytoplankton and bacteria, it is desirable to learn to what extent and by which mechanisms the metabolic activity of heterotrophic bacteria regulate carbon and nutrient cycling through the microbial loop.

Monitoring the actual rates of the plethora of metabolic pathways active in microbial communities directly *in situ* is currently not feasible, but nucleotide sequencing-based methods, in particular metatranscriptomics, can indicate which microbial metabolisms are actively transcribed. Metatranscriptomics has hence become a widely used tool to provide detailed insights into the genetic underpinnings of metabolic responses within communities both in natural environments and controlled experiments [9–12]. The complexity of gene regulation observed in communities consisting of many thousands of individual populations is, however, daunting. For the purpose of eventually using transcript profiling as a proxy for metabolic activity in complex natural communities, zooming in to compare gene expression patterns in isolates of environmentally relevant microbial taxa could be useful.

With the aim of charting the possibility of using transcriptional activity of bacterial isolates as living sensors for the flow of nutrients in the ecosystem, we exposed two Baltic Sea model bacteria to DOM derived from axenic cultures of *Prorocentrum minimum*, a dinoflagellate that forms major blooms in the spring and autumn in the Baltic Sea. The bacterial isolates–*Polaribacter* sp. strain BAL334 (*Flavobacteriaceae*, *Bacteroidota*) and *Brevundimonas* sp. strain BAL450 (*Caulobacteraceae*, *Alphaproteobacteria*)–were selected to compare responses of bacteria with different evolutionary histories. Furthermore, since previous studies of phytoplankton extracellular DOM release have suggested that phytoplankton secrete different compounds during early and late growth phases [13–17], the DOM was harvested both from dinoflagellates growing actively and in stationary phase. This allowed characterization of potential differences in bacterial responses to DOM released during exponential and senescence phases of phytoplankton blooms.

## Materials and methods

### Cultivation of axenic *Prorocentrum minimum*

An axenic culture of the dinoflagellate *Prorocentrum minimum* strain CCMP1329 was obtained from the Provasoli-Guillard National Center of Marine Algae and Microbiota (CCMP; https://ncma.bigelow.org/). 5 mL of inoculum of *P*. *minimum* CCMP1329 was transferred and cultivated in axenic conditions in 6 replicates using acid-washed Erlenmeyer flasks (2 L) containing 1.3 L of L1 medium [18], prepared using 0.2 μm membrane filters (Supor®, Pall Corporation) and artificial seawater (30 practical salinity units, prepared from Sea Salts; Sigma). The cultures were placed in 20°C with photosynthetically active radiation (PAR) of 83–101 μmol photon m^-2^ s^-1^ in light:dark cycles of 13:11 h and bubbled with filtered air provided by an inhouse air system. To follow the growth of the cultures, chlorophyll *a* concentrations were measured regularly by collecting 1 mL of culture on 25 mm glass microfiber filters (GF/C, Glass Microfiber Binder Free, Whatman), followed by chlorophyll *a* ethanol extraction according to [19].

### Collection of DOM

DOM from *P*. *minimum* CCMP1329 was collected from three of the cultures in the exponential growth phase (~15 days after inoculation) and from two of the cultures in stationary phase (~31 days), hereafter referred to as DOM_exp and DOM_sta, respectively. DOM from the exponential growth phase was retrieved as follows. Phytoplankton cells were gently removed by first filtering through an acid-washed 3.0 μm polycarbonate filter (GSV, Life Science) and then through an acid-washed 0.22 μm polycarbonate filter (GSV, Life Science), using a Sterifil 47 mm filter holder (Merck Millipore). DOM collected in stationary growth phase was obtained by first centrifuging the cultures in acid-washed 50 mL Falcon tubes for 10 min at 3000 g (to prevent filters from clogging); the supernatant was then filtered through an acid-washed 0.22 μm polycarbonate filter (GSV, Life Science) using a Sterifil 47 mm filter holder (Merck Millipore). The flow-through liquid was transferred into an acid-washed 10 L polycarbonate (PC) bottle and mixed before samples for dissolved organic carbon (DOC) concentration and microscope samples were taken (see below for detailed information of the sampling procedure). Finally, the DOM was aliquoted into 1 L acid-washed PC bottles and stored at -80°C until further proceedings.

### Bacterial isolates and culture conditions

The flavobacterium *Polaribacter* sp. strain BAL334 (hereafter referred to as *Polaribacter* BAL334) and alphaproteobacterium *Brevundimonas* sp. strain BAL450 (hereafter referred to as *Brevundimonas* BAL450) were isolated from surface water (2 m depth) at the Linnaeus Microbial Observatory (LMO) in the Baltic Sea (N 56° 55.8540’, E 17° 3.6420’) during 2012. Seawater was spread on Baltic Zobell agar plates containing a mixture of 5 g bacto peptone, 1 g yeast extract and 15 g bacto agar per L of sterile Baltic Sea water (i.e. a mix of 750 ml seawater and 250 ml MilliQ water). Bacterial colonies were transferred into 1 mL of Baltic Zobell medium (i.e. mixture of 5 g bactopeptone and 1 g yeast extract per L of sterile Baltic Seawater) and preserved in glycerol (25%, final concentration) in -80°C.

### DNA extraction and genome sequencing

To identify the bacteria, DNA from the isolates were extracted using the E.Z.N.A. Tissue DNA kit (Omega bio-tek, USA) following the manufacturer’s protocol for extraction of cultured cells in suspension. For identification of the isolates, bacterial 16S rRNA genes were PCR amplified using the primers 27F and 1492R [20] at a final concentration of 10 μM with the following PCR thermal cycling program: 95°C for 2 min; 30 cycles of 95°C for 30 s, 50°C for 30 s, and 72°C for 45 s; and 72°C for 7 min. E.Z.N.A. The Cycle-Pure Kit (Omega bio-tek, USA) was used for cleaning the PCR product following the manufacturer’s spin protocol instruction. Samples were sent for Sanger sequencing at Macrogen Europe, Amsterdam, Netherlands. The partial 16S rRNA gene sequences have been deposited in GenBank with the following accession numbers: KM586879 (*Polaribacter* BAL334) and KM586934 (*Brevundimonas* BAL450).

Genome sequences from the isolates *Polaribacter* BAL334 and *Brevundimonas* BAL450 were obtained by sequencing the extracted genomic DNA using the Illumina HiSeq 2500 system (PE 2x125bp) at SciLifeLab, Solna, Sweden. The quality of sequences was checked with FastQC (version 0.11.5) [21] and MultiQC (version 1.4) [22], adaptors were removed with cutadapt (version 1.12) [23] and Sickle (version 1.33) [24] was used to trim sequences based on quality score. Assembly was performed using Megahit (version 1.1.2) [25] and annotation with the Rapid Annotation using Subsystem Technology (RAST) server [26, 27]. The genomes are available in the RAST database SEED viewer (https://rast.nmpdr.org/seedviewer.cgi) with identities 6666666.325781 (https://rast.nmpdr.org/seedviewer.cgi?page=Organism&organism=6666666.325781) and 6666666.325780 (https://rast.nmpdr.org/seedviewer.cgi?page=Organism&organism=6666666.325780) for *Polaribacter* BAL334 and *Brevundimonas* BAL450, respectively, using the guest account (user login “guest”, password “guest”).

### Experimental setup and spiking of DOM

All cultivations of the two studied bacteria for the experiments, as well as the experiments themselves, were carried out at room temperature and standard laboratory light conditions (fluorescent lamps). Bacterial isolates were grown on Baltic Zobell agar plates for 3–4 days after being transferred from the -80°C freezer. Subsequently, they were inoculated into an acid-washed 100-mL glass bottle containing 20 mL Baltic Zobell medium, and were allowed to grow for 28 hours (*Polaribacter* BAL334) and 54 hours (*Brevundimonas* BAL450) on a Unimax 2010 orbital shaker (Heidolph) at 120 rpm. 2 mL of bacterial culture *Polaribacter* BAL334 (reaching an optical density [OD_600_] of 0.39) and 0.5 mL of bacterial culture *Brevundimonas* BAL450 (reaching OD_600_ of 0.57) were transferred to acid-washed 2 (L) glass bottles containing 300 mL fresh Baltic Zobell medium. *Polaribacter* BAL334 was grown into early stationary phase at 80 rpm (38 hours; and OD_600_ 0.20) and *Brevundimonas* BAL450 bacterial cultures was grown into early stationary phase (40 hours; OD_600_ 1.3) at 120 rpm. To reduce nutrient concentrations and adapt bacterial cells to starvation, bacterial cells were centrifuged at 4000 rpm for 7 min, supernatants were discarded and cell pellets were washed by adding 1 volume of sterile artificial Baltic seawater (7 PSU, prepared from Sea Salts; Sigma). Cells were resuspended in artificial Baltic seawater and the procedure was repeated twice more.

To start the experiment, 30 mL (*Polaribacter* BAL334) and 14.5 mL (*Brevundimonas* BAL450) of resuspended bacterial cells were divided into each of nine acid-washed (1 L) glass bottles containing 700 mL artificial seawater (7 practical salinity units [PSU], prepared from Sea Salts; Sigma). The different volumes were to start the experiment with similar bacterial numbers (2–7 x 10^6^ cells ml^-1^), and were based on OD measures in the washed cell suspensions (see previous paragraph). Subsequently, after 1.5 hours, three of the bottles were spiked with 67 mL DOM_exp and three with 14 mL DOM_sta to obtain an enrichment with DOC corresponding to ~50 μM carbon (final concentration). To minimize potential effects from the medium used for culturing *P*. *minimum* between the treatments, 53 mL of L1 medium were added to the DOM_sta bottles. Three bottles serving as controls were spiked with 67 mL of L1 medium. Adding a total of 67 mL L1 medium (30 PSU) to all experimental bottles and the controls resulted in a decrease in salinity of ~0.5 PSU to a salinity of ~6.5 PSU. The salinity at the LMO station from where the bacteria were isolated typically varies between 6.4 and 7.6 PSU on a yearly basis [28]. Samples for determination of DOC concentrations and bacterial abundances were taken as described below.

### Determination of DOC concentrations, bacterial abundance, OD and purity of cultures using microscopy and cultivation

Samples for DOC concentrations were collected 1 hour after DOM spiking by filtering 30 mL of sample through 0.2 μm acrodisc supor syringe filters 32 mm, into a 60 mL TC flask (Sarstedt) using a 50 ml plastic syringe (NORM-JECT). Samples were then acidified by addition of 448 μl 1.2 M HCl. DOC concentrations were calculated as non purgeable organic carbon (using high temperature catalytic oxidation followed by NDIR detection of the gaseous CO_2_), analyzed on the high-temperature carbon analyzer Shimadzu TOC-5000 at Umeå Marine Science Centre, Umeå, Sweden. Bacterial abundance (BA) samples were taken in triplicates from each replicate 1 hour after DOM spiking, by fixing the sample with paraformaldehyde at a final concentration of 1%. Samples were then frozen at -80°C until determined by using the flow cytometer Cube 8 (CyFlow®) according to protocol in [29]. Optical density at 600 nanometer (OD_600_) was measured with a Beckman DU®640 spectrophotometer. To ensure axenic conditions (i.e. exclusion of bacterial contamination), 1 mL of algae cultures and samples from experiments were fixed with 1% paraformaldehyde, stained with 0.02% SYBR gold (final concentration) and filtered onto 0.2 μm, 25 mm black polycarbonate filters (Millipore). The filters were analyzed by epifluorescence microscope observation before and after the cultivations/experiments. Additionally, aliquots from bacterial and phytoplankton cultures were spread on Zobell agar plates for investigation of potential contamination.

### RNA sampling, extraction and sequencing

One hour after DOM spiking, seawater samples for RNA were fixed by addition of an ethanol:phenol mix (5% phenol in absolute ethanol) in a 10:1 proportion [30]. The time of exposure of bacteria to the DOM sources was kept short to enable detection of the near-immediate response of the bacteria to the new organic matter field that they got exposed to (see e.g., [31]). The fixed samples were then filtered through Durapore 0.2 μm, 47-mm membrane filters GV (Merck Millipore). Filters were folded and transferred into clean nucleotide-free collection tubes and stored at -80°C until extraction. Extraction of RNA was performed using the RNeasy mini kit (Qiagen). Briefly, bacterial cells were lysed by cutting membrane filters into smaller pieces and placing them in nucleic acid free microfuge tubes containing RLT Buffer (with added ß-Mercaptoethanol 1:100) and 1.5 gram 200 μm Low Binding Zirconium Beads (OPS diagnostics, USA). Thereafter, cell lysis, RNA purification, on column DNase digestion and RNA elution were performed following the manufacturer’s instructions. Total RNA was DNase treated using the TURBO DNA-free Kit (ThermoFisher Scientific) and quality checked on agarose gel. Ribosomal RNA was depleted using RiboMinus Transcriptome Isolation Kit and RiboMinus Concentration Module (ThermoFisher Scientific) and mRNA was amplified using the MeassageAmp II-Bacteria RNA Amplification Kit (ThermoFisher Scientific) according to manufacturer’s instructions. RNA sequencing was done at the at SciLifeLab, Solna, Sweden. Raw sequence reads are available at NCBI’s Sequence Read Archive under the BioProject PRJNA678611 (https://www.ncbi.nlm.nih.gov/bioproject/PRJNA678611).

### Bioinformatics and statistical analysis

RNA sequence reads were quality trimmed with Sickle (version 1.33) [24]. Ribosomal RNA (rRNA) was bioinformatically filtered by mapping the reads against an in-house database containing rRNA sequences from marine microbes using ERNE [32] version 2.1.1. The remaining reads were mapped to the genomes of our two studied bacteria with Bowtie 2 [33]. This resulted in between 92,655 and 329,457 mRNA sequence reads per sample mapping to coding DNA sequence (CDS) features. EdgeR [34] was used to determine significantly differentially expressed genes between treatments and controls, after removal of genes with a total count of less than five reads across all samples. As significance level, we applied a false discovery rate (FDR) of <5% (rather than a chosen minimal level of fold change) since this imposes comparably strict requirements both on transcripts with low abundance and those with high abundance in the data set. We chose statistical testing of treatment responses to the respective controls, as we have done previously [35], to avoid simultaneously making multiple statistical inferences and because the controls for the two studied bacteria were the same (fresh dinoflagellate culture medium only). EdgeR was also used to retrieve normalized counts per million (CPM) estimates.

## Results

### Growth of *Prorocentrum minimum*

**Fig 1:**
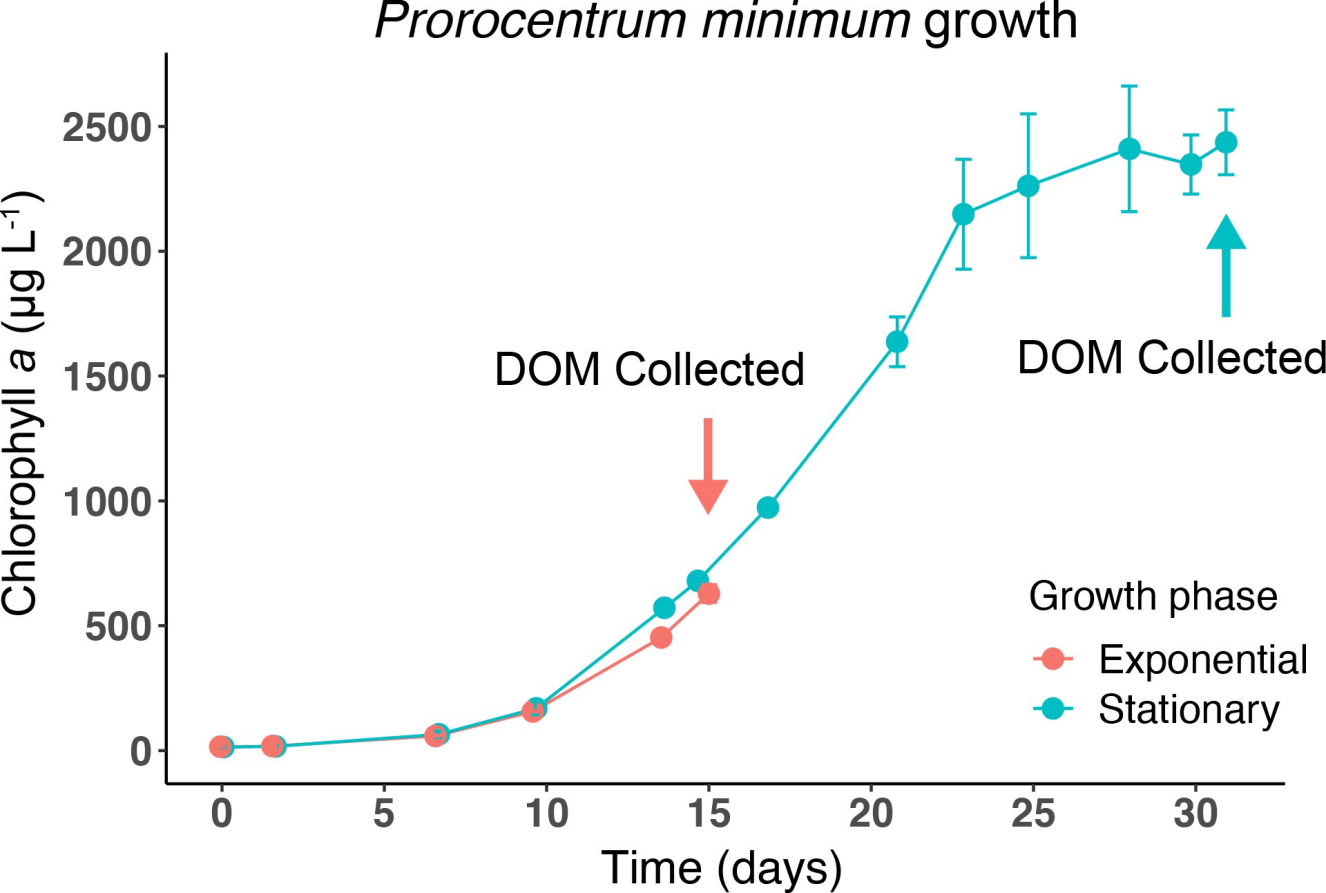
Growth of axenic *P*. *minimum* cultures in L1 medium for collection of dissolved organic matter from different growth phases. Red line denotes growth of cultures harvested for DOM in exponential phase, and blue line shows growth of cultures harvested in stationary phase. Chlorophyll *a* concentrations were monitored as a proxy for biomass. Error bars denote the standard deviations of triplicates for exponential phase cultures and duplicates for stationary phase; when not visible, error bars are within symbols.

The dinoflagellate *Prorocentrum minimum* was grown to obtain the DOM sources for the experiments. It followed a sigmoid growth curve with a lag phase of about 10 d. Thereafter, it entered an exponential growth phase. DOM was collected during active growth (day 15) at a chlorophyll *a* concentration of 629 ± 35 μg/L. After 31 d the samples for stationary phase DOM were retrieved at a chlorophyll *a* concentration of 2436 ± 130 μg/L (**Fig 1**).

### Bacterial abundance

In *Polaribacter* BAL334 cultures spiked with DOM from exponential and stationary phase dinoflagellate cultures, bacterial abundance reached 6.6 ± 0.6 and 6.4 ± 0.6 x 10^6^ cells ml^-1^ (mean ± standard deviations, n = 3), respectively. This was nearly a doubling compared to the control cultures where 3.8 ± 0.7 x 10^6^ cells ml^-1^ were recorded. Bacterial abundance in the *Brevundimonas* BAL450 cultures spiked with exponential phase DOM increased to 3.7 ± 0.4 x 10^6^ cells ml^-1^, and further increased to 4.6 ± 0.4 x 10^6^ cells ml^-1^ with stationary phase DOM. Control culture cell abundance was 1.7 ± 0.4 x 10^6^ cells ml^-1^.

### Brief description of the *Polaribacter sp*. BAL334 and the *Brevundimonas sp*. BAL450 genomes

*Polaribacter* sp. BAL334 (*Bacteroidota*) and *Brevundimonas* sp. BAL450 (*Alphaproteobacteria*) have similar genome sizes at ~3.3 Gb and ~3.2 Gb, respectively. The *Polaribacter* sp. BAL334 genome contains 2880 putative open reading frames (ORFs), of which 1021 (35.5%) have a SEED annotation, whereas 757 (26.3%) have a functional annotation but were not represented in SEED. The remaining 1102 ORFs (38.2%) were annotated as hypothetical proteins. The *Brevundimonas* sp. BAL450 genome encodes 3001 ORFs. Of these, 1269 (42.3%) have a SEED annotation, and 732 (24.4%) were not included in SEED but were functionally annotated; the remaining 1000 ORFs (33.3%) were annotated as hypothetical proteins.

**Fig 2:**
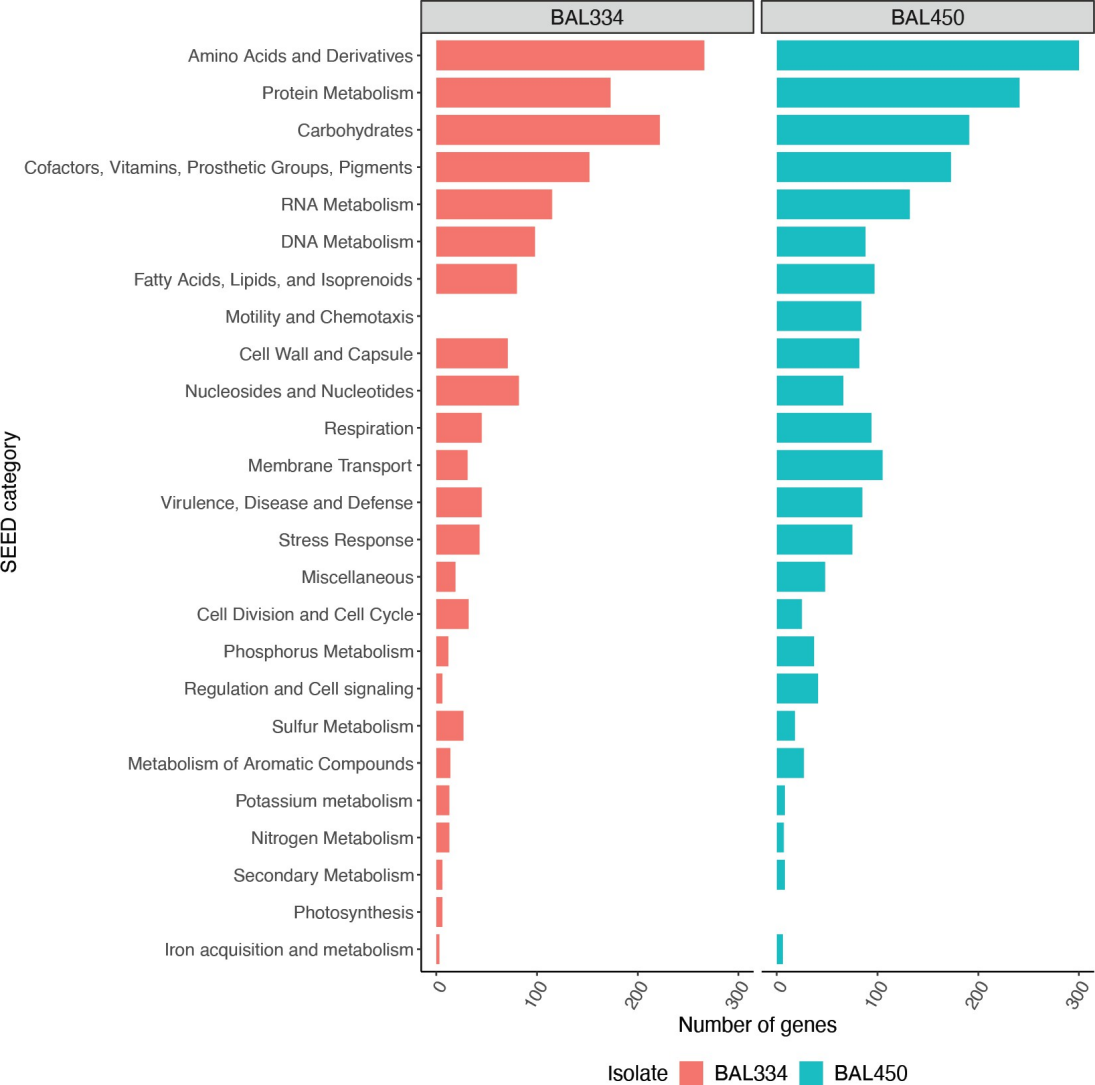
Comparison of number of genes in the genomes of the two studied bacteria. Number of genomically encoded genes in top level SEED categories. “BAL334” and “BAL450” refers to *Polaribacter* BAL334 and *Brevundimonas* BAL450, respectively.

As expected due to their role in central metabolism, dominant SEED categories (the highest level in the SEED hierarchy) in the genomes of both isolates were *Amino Acids and Derivatives* (up to 300 genes), *Carbohydrates*, *Protein Metabolism*, and the category *Cofactors*, *Vitamins*, *Prosthetic groups*, *Pigments* (**Fig 2**). Although the genome sizes of the two bacteria was comparable, *Polaribacter* BAL334 had a higher number of genomically encoded genes devoted to the categories *Carbohydrates* (222 genes, compared to 191 in *Brevundimonas* BAL450) and *Sulfur metabolism* (37 versus 18 genes) and *Photosynthesis*; the latter reflecting that only *Polaribacter* BAL334 has the proteorhodopsin gene encoded in its genome. In contrast, the category *Motility and Chemotaxis* was restricted to *Brevundimonas* BAL450 (84 genes), reflecting the lack of flagellar motility in *Polaribacter* BAL334 (**Fig 2**). Moreover, the number of genes in the category *Membrane Transport* (105 genes) was over threefold higher in *Brevundimonas* BAL450.

### Messenger RNA sequencing outcome

Sequenced bacterial mRNAs sampled 1 h after exposure to the different DOC sources mapped to 2742 ORFs in *Polaribacter* BAL334 and 2984 ORFs in *Brevundimonas* BAL450, representing 95% and 99% of putative protein coding genes in the genomes of the two isolates, respectively. The SEED annotated genes (with full functional hierarchies allowing higher level metabolic analyses; 995 and 1228 respectively in *Polaribacter* BAL334 and *Brevundimonas* BAL450), attracted 36–38% of the mRNA reads in *Polaribacter* BAL334 and 56–58% of the reads in *Brevundimonas* BAL450. The higher SEED annotation levels in the transcriptome of *Brevundimonas* BAL450 most likely reflected that knowledge of *Proteobacteria* genetics is generally higher than for *Bacteroidota*.

### Overview of transcriptional differences between the two bacteria

**Fig 3:**
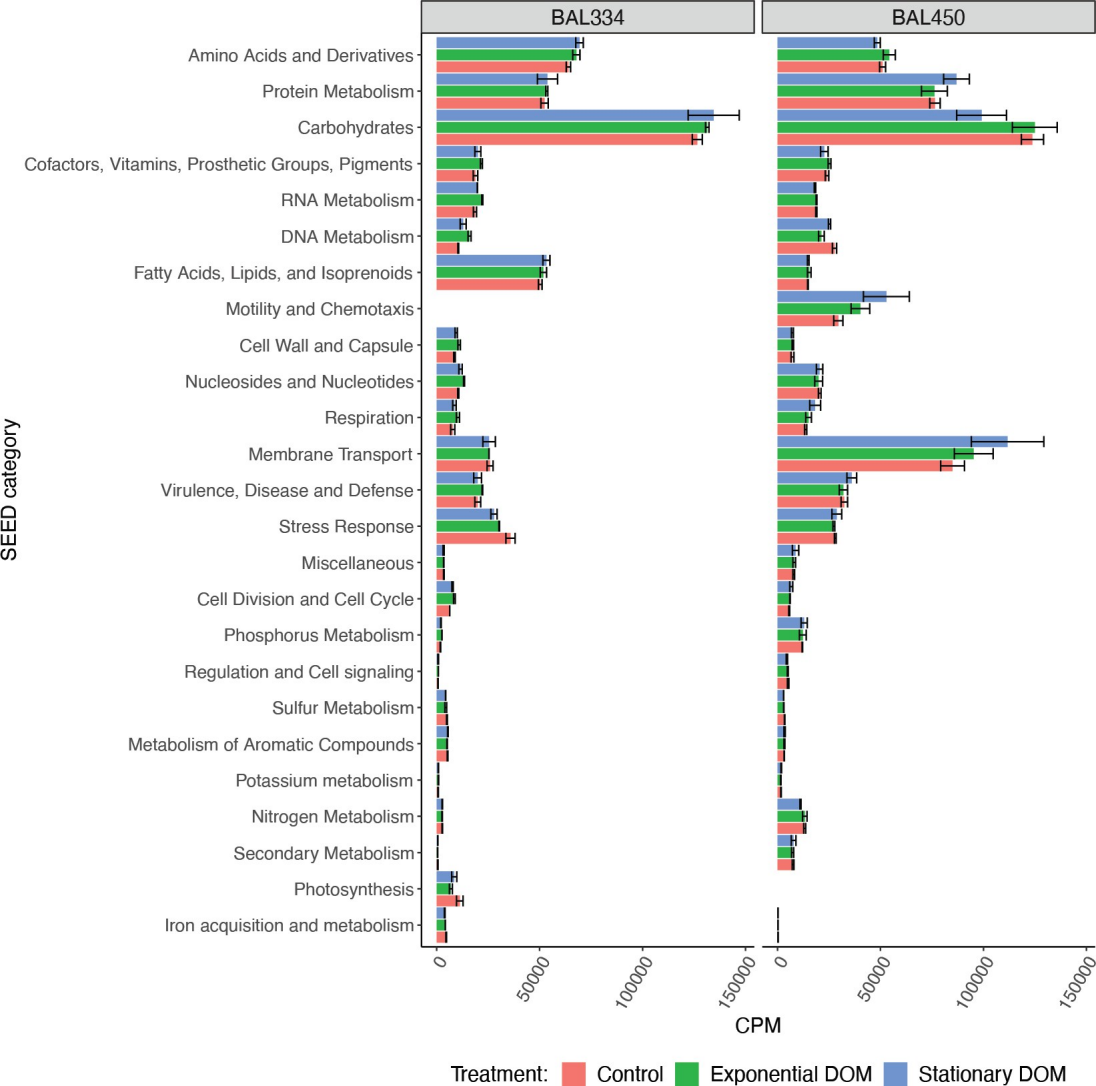
Comparison of the relative expression levels in the two studied bacteria exposed to distinct DOM. Relative gene expression responses to enrichment with DOM collected from axenic exponential and stationary phase dinoflagellate cultures, and to control enrichments with only dinoflagellate L1 medium. “BAL334” and “BAL450” refers to *Polaribacter* BAL334 and *Brevundimonas* BAL450, respectively. Error bars denote standard deviations for triplicates per treatment.

Comparison of the transcriptomes showed that the relative expression level in the category *Membrane Transport* was threefold higher in *Brevundimonas* BAL450 than in *Polaribacter* BAL334 (~97,000 versus ~26,000 CPM) (**Fig 3**). *Brevundimonas* BAL450 also had higher expression in the two categories *Nitrogen Metabolism* and *Phosphorus Metabolism*. In contrast, expression in the category *Fatty Acids*, *Lipids*, *and Isoprenoids*, was ~3-fold higher in *Polaribacter* BAL334 than in *Brevundimonas* BAL450 (~52,000 versus ~15,000 CPM) (**Fig 3**).

**Fig 4:**
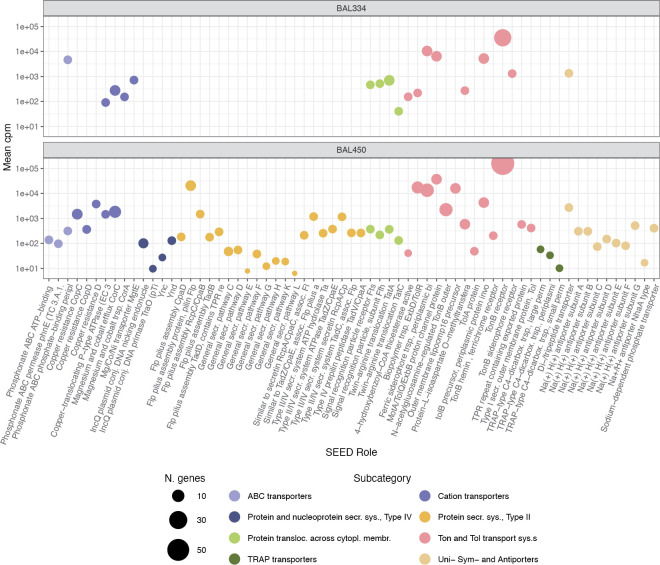
Comparison of relative expression levels of genes in the SEED category *Membrane Transport* in the two studied bacteria. Colors of circles denote transporter types. The size of circles represents the number of paralogs (defined as ORFs with the same name) in each of the genomes of the two bacteria. Y-axes shows the expression in counts per million (CPM) for the DOM treatments and the controls.

The major differences between the isolates in expression of the SEED categories *Membrane Transport* and *Fatty Acids*, *Lipids*, *and Isoprenoids* warranted closer inspection. In *Membrane Transport* (**Fig 4**), we identified many more expressed genes in *Brevundimonas* BAL450 (64 paralog groups) than in *Polaribacter* BAL334 (18 paralog groups). Moreover, in *Brevundimonas* BAL450, these genes were distributed in a broader variety of transporter subsystems (the second level in the SEED hierarchy; roughly corresponding to “pathways” in other annotation databases). In both isolates, transporter expression was dominated by the *Ton and Tol transport* subsystem, both in relative expression levels and the number of expressed genes. *TRAP transporters* and *Uni-*, *Sym- and Antiporters* were well represented in *Brevundimonas* BAL450 but were absent from *Polaribacter* BAL334 (**Fig 4**).

**Fig 5:**
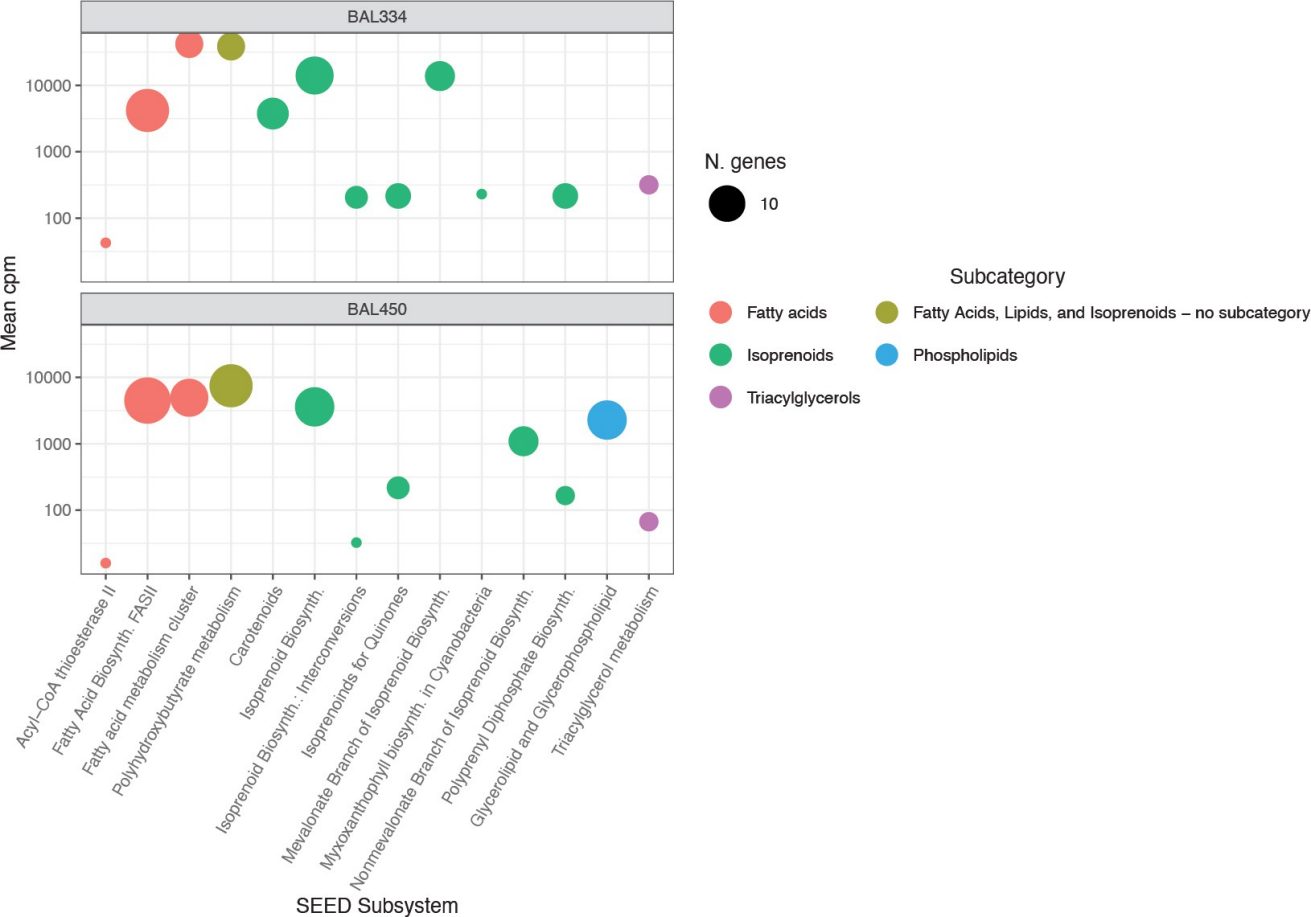
Analysis of gene expression in SEED subsystems of the *Fatty Acid*, *Lipids and Isoprenoids* category. Colors of circles denote SEED subcategories. The size of symbols represents the number of genes expressed in each subsystem. Y-axes shows the expression in counts per million (CPM) for the two DOM treatments and the controls.

The higher expression in the category *Fatty Acids*, *Lipids*, *and Isoprenoids*, in *Polaribacter* BAL334 was primarily due to differences in the subsystems *Fatty Acid metabolism* and *Polyhydroxybutyrate metabolism* (PHB) (**Fig 5**). These two subsystems contained few expressed genes (five in both subsystems in *Polaribacter* BAL334; 11 and 15, respectively, in *Brevundimonas* BAL450; **Fig 5**). In fact, two of the three most abundantly expressed genes in the *Fatty Acids*, *Lipids*, *and Isoprenoids* category (*3-hydroxyacyl-CoA dehydrogenase* and *3-ketoacyl-CoA thiolase*) are shared between the subsystems, while the third (*Acetyl-CoA acetyltransferase*) occurred only in PHB. Still, expression of these genes differed substantially between the isolates, with higher levels in *Polaribacter* BAL334 (**S1 Table**). Moreover, *Polaribacter* BAL334 had higher expression of genes involved in isoprenoid synthesis (**Fig 5**), in line with isoprenoids being precursors for carotenoids, which are likely responsible for the vividly orange color of *Polaribacter* BAL334 colonies.

### Statistical differences in bacterial transcription between exponential and stationary phase dinoflagellate DOM

**Fig 6:**
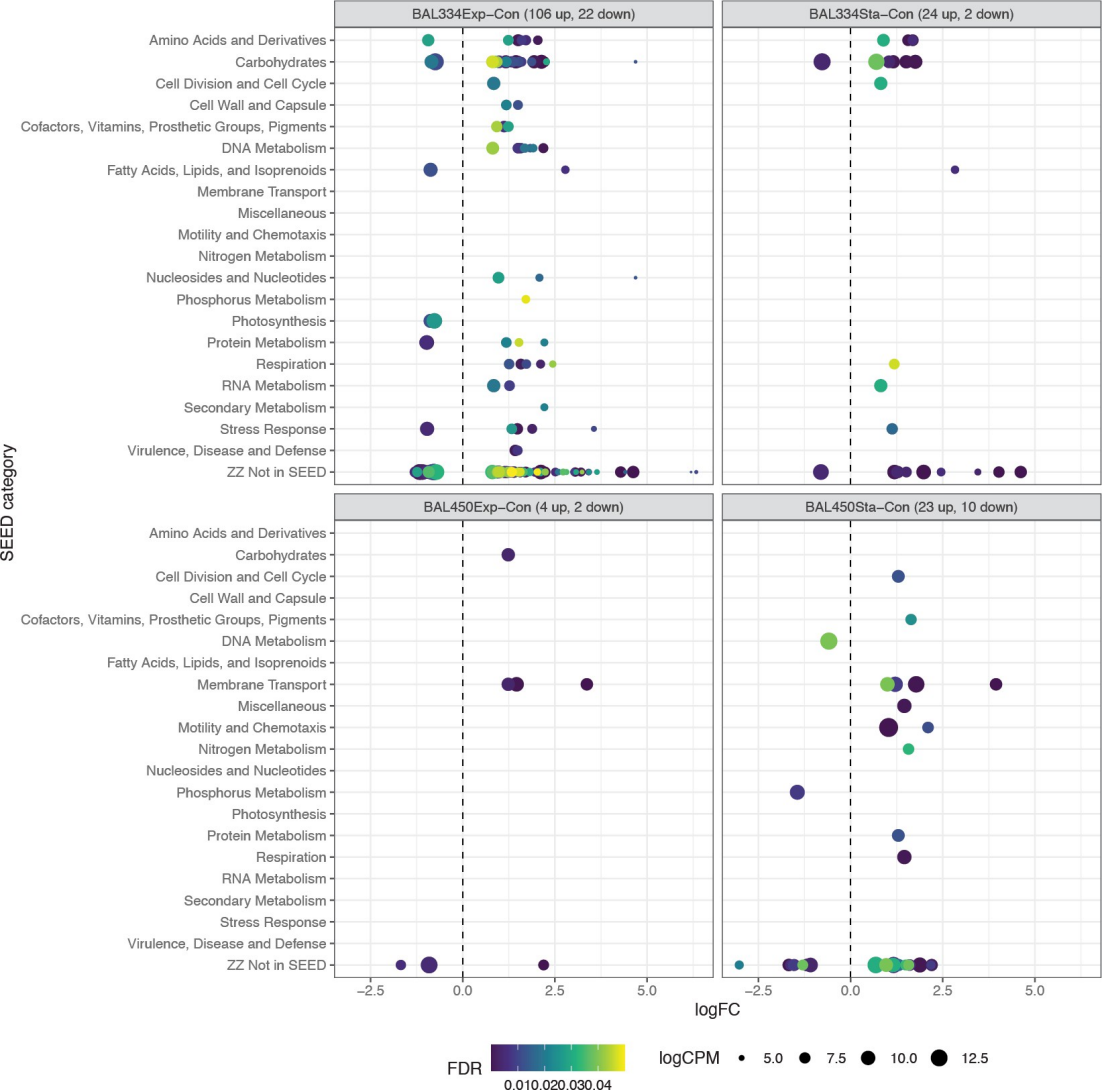
Influence of DOM from different growth phases of the dinoflagellate *Prorocentrum minimum* on statistically significant differences in gene expression between the two marine bacteria. Transcripts were defined as statistically significantly differentially abundant based on EdgeR analyses with an FDR ≤ 0.05. Each circle represents one gene and the circle size shows the calculated expression in logCPM; note that a gene can occur in more than one SEED category (i.e. there can be some more circles in the plots than the number of genes given in the plot headers). Genes with significantly lower expression in controls compared to treatments are indicated with negative logarithmic fold change (logFC) values. BAL334Exp-Con and BAL334Sta-Con refers to *Polaribacter* BAL334 with DOM from exponential (Exp) and stationary (Sta) phase DOM compared to controls (Con). BAL450Exp-Con and BAL450Sta-Con refers to *Brevundimonas* BAL450 with DOM from exponential (Exp) and stationary (Sta) phase DOM compared to controls (Con).

The EdgeR analysis to determine which expressed genes were significantly more (denoted “up”) or less (denoted “down”) abundant in the bacterial transcriptomes of the DOM-enriched samples than in controls was partitioned into four contrasts, considering each of the bacterial isolates and each of the DOM pools from the dinoflagellate relative to the control samples that did not receive any DOM. *Polaribacter* BAL334 enriched with DOM from exponential phase dinoflagellates compared to the control (hereafter, *Polaribacter* BAL334Exp-Con contrast) contained by far the highest number of differentially expressed genes (total of 128 genes; 106 up and 22 down; *false discovery rate <5%*) (**Fig 6**). The treatment with DOM from stationary phase dinoflagellates (hereafter, *Polaribacter* BAL334Sta-Con) resulted in much fewer differentially expressed genes (24 genes up and two down). In *Brevundimonas* BAL450, on the other hand, it was the DOM from stationary phase dinoflagellates (hereafter, *Brevundimonas* BAL450Sta-Con) that induced more differentially expressed genes (23 genes up and ten down) than the exponential phase DOM (hereafter, *Brevundimonas* BAL450Exp-Con): four genes up and two down (**Fig 6**). None of the significantly differentially expressed genes were shared between *Polaribacter* BAL334 and *Brevundimonas* BAL450 (**S2 Table**). In both bacterial isolates the majority of expressed genes were not included in any SEED category (denoted “Not in SEED” in **Fig 6**).

**Fig 7:**
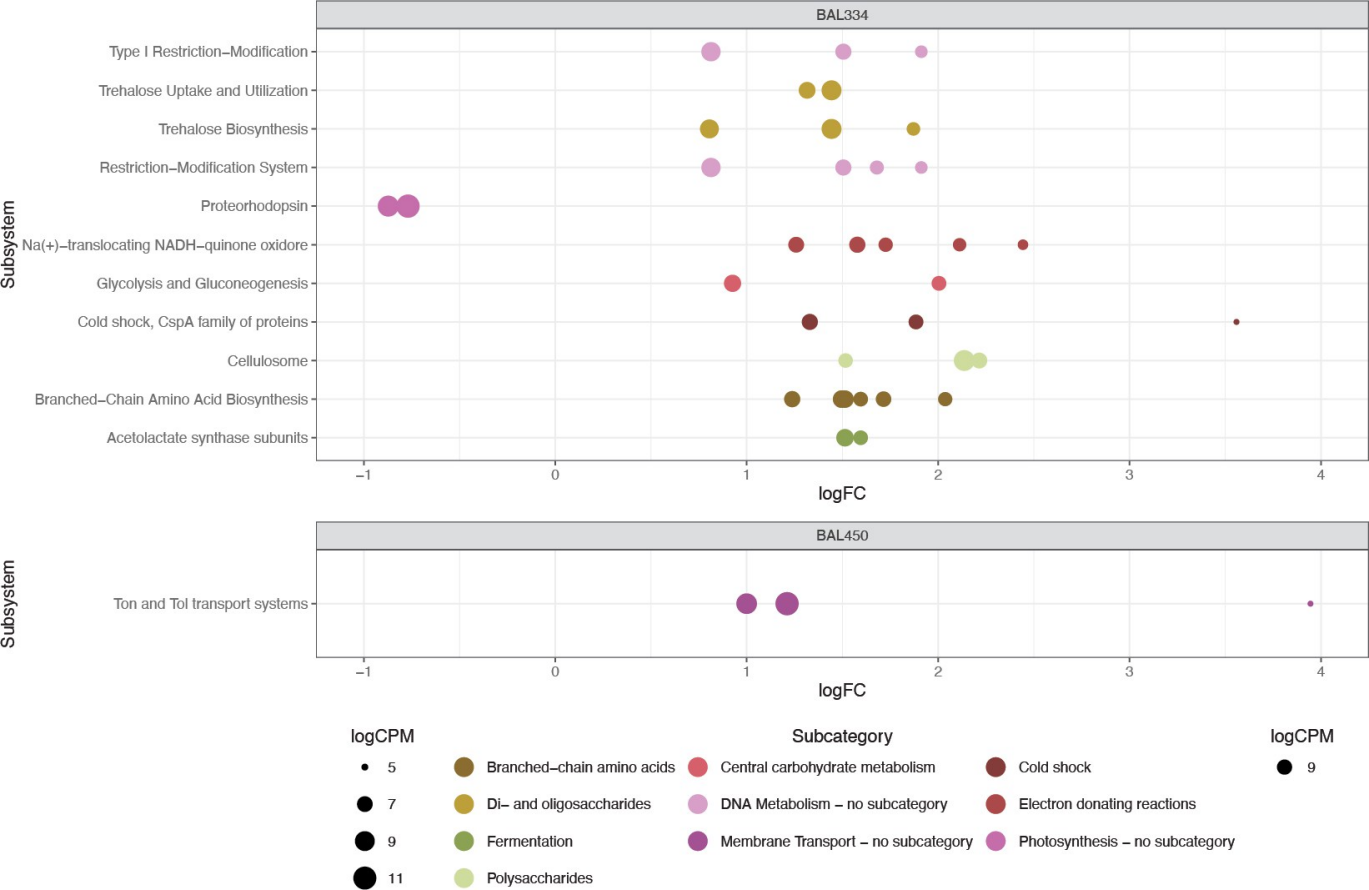
Subsystems in *Polaribacter* BAL334Exp-Con containing at least two significantly differentially expressed genes. SEED subsystem is shown on Y-axis and SEED category is shown in each plot title. The X-axis shows the number of genes whose expression is significantly more (positive value) or less (negative value) abundant compared with controls. Circle size represents the sum of logCPM.

Out of the 128 differentially expressed genes, 104 genes were unique to the *Polaribacter* BAL334 exponential phase dinoflagellate DOM. Among the twelve SEED subsystems that had at least two differentially expressed genes compared to the control (**Fig 7 and S2 Table**), the *Carbohydrates* category stood out by being represented by five subsystems that increased in expression in the treated samples, three of which were also highly expressed: *Trehalose Uptake and Utilization*, *Trehalose Biosynthesis*, and *Cellulosome* (**Fig 7 and S2 Table**). Several of the more abundantly expressed genes in these subsystems encoded enzymes involved in the utilization of starch, including a gene for SusC that is an outer membrane protein involved in starch binding and eight genes involved in starch degradation to trehalose/maltose and the potential modification of these sugars (e.g. genes for alpha-amylase and maltose/trehalose phosphorylase) (**S2 Table**). Moreover, among the 67 transcribed genes not annotated in SEED, at least twelve are tentatively involved in carbohydrate metabolism, including an alpha-glucosidase gene that was up in exponential phase dinoflagellate DOM and two highly expressed pullulanases that are exoenzymes involved in the degradation of the polysaccharide pullulan (both were significantly up also in the DOM from stationary phase dinoflagellate DOM) (**S2 Table**).

In the *Amino Acids and Derivatives* category, the *Branched-Chain Amino Acid Biosynthesis* subsystem contained six genes involved in isoleucine synthesis. In the *DNA Metabolism* category, three type I restriction-modification system genes were detected, shared by two subsystems plus a type III restriction-modification system gene found only in the *Restriction-Modification System* subsystem (**Fig 7 and S2 Table**). Only two significantly differentially abundant genes were unique to the contrast *Polaribacter* BAL334Sta-Con, encoding 2-isopropylmalate synthase (*Amino Acids and Derivatives*) and a highly abundant *susC* paralog distinct from the one that was abundant in the exponential phase DOM.

Finally, only the *Proteorhodopsin* subsystem (in SEED category *Photosynthesis*) had multiple genes with relative expression levels that were significantly higher in the controls (seen as negative value of differentially expressed genes in **Fig 7**). These were the proteorhodopsin gene and a phytoene dehydrogenase, which is involved in the synthesis of the rhodopsin chromophore retinal. Both were abundantly expressed with logCPM values of 11.1 and 9.6, respectively (i.e. ~0.2% and 0.08% of total transcripts).

Among the 32 significant genes that were unique in the *Brevundimonas* BAL450Sta-Con contrast, the Ton and Tol transport system (*Membrane Transport*) was the only subsystem that contained at least two differentially expressed genes (**Fig 7 and S2 Table**). Two of the three genes were annotated as *TonB-dependent receptors*, and were shared between the exponential and stationary phase DOM contrasts with controls. A particularly highly expressed TonB transport system gene (logCPM of 10.7) was annotated as encoding a ferric siderophore periplasmic binding protein, which was unique to the stationary phase DOM (**S2 Table**). In contrast, the *N-acetylglucosamine (NAG)-regulated TonB-dependent outer membrane receptor* was unique to the treatment with exponential phase DOM, where also a beta-glucosidase gene was found (i.e., BAL450Exp-Con contrast; **Fig 7 and S2 Table**).

## Discussion

We investigated how representatives of two major taxa in the marine environment–*Flavobacteriia* and *Alphaproteobacteria*–react to DOM produced by dinoflagellates in different growth phases. These reactions partly reflected differences in genomically encoded functional capacity, but also that each of the isolates changed their allocation of relative transcriptional investment in certain metabolic functions. This change in transcription coincided with roughly a doubling in abundance following a single hour of DOM exposure. In agreement with the large phylogenetic distance between the two isolates, there were striking differences in the gene expression responses to DOM between the two isolates. This suggests pronounced differences in resource utilization between these marine bacteria, indicating a potential for resource partitioning at the level of major metabolic categories.

### Comparison of bacterial gene expression to dinoflagellate DOM from different growth phases

As deduced from the number of genes that differed significantly in expression, we found that *Polaribacter* BAL334 (total 154 genes; 5.6% of genome) showed a much stronger response to DOM enrichment than *Brevundimonas* BAL450 (total 39 genes; 1.3% of genome). The responsiveness of *Polaribacter* BAL334 is in line with findings from the marine environment that *Flavobacteriia* have a pronounced ability to utilize organic matter produced during periods of phytoplankton blooms [36, 37]. Moreover, it is noteworthy that the majority of significantly differentially expressed genes in *Polaribacter* BAL334 were observed in treatments with DOM from exponential phase dinoflagellates, whereas for *Brevundimonas* BAL450 most significantly expressed genes were found with stationary phase DOM. The latter observation was consistent with the finding that BAL450 reached higher bacterial abundances when incubated with stationary phase DOM. These findings are in line with recent elegant evidence that DOM from phytoplankton exudates and lysates (from both a diatom and a cyanobacterium species) is utilized by different taxa in natural bacterioplankton and by means of distinct molecular mechanisms [5]. Our work contributes to the understanding of how bacteria differentially utilize qualitatively different phytoplankton DOM by showing that bacterial populations have diverged in their adaptation to utilize DOM produced and released by dinoflagellates during active growth compared to stationary phase.

A notable observation in the *Polaribacter* BAL334 transcriptional response to dinoflagellate DOM was the high expression of two *susC* paralogs, putatively annotated as transporters of starch, that were highly expressed and that showed different expression patterns between the DOM treatments. This contrasted strongly with the much reduced gene expression in the DOM treatments of a TonB dependent receptor for sucrose. SusC is a component of the polysaccharide utilization loci [PULs] widespread in *Bacteroidota* that mediate the initial binding/transport of polysaccharides in a TonB dependent manner [38]. Interestingly, one *susC* paralog was highly expressed in DOM from both exponential and stationary phase dinoflagellates, but the second was highly expressed only by bacteria exposed to stationary phase DOM. These findings provide experimental verification of genome analysis results [39, 40] on the importance of polysaccharides to *Flavobacteriaceae* species. Also for *Brevundimonas* BAL450 the transcriptional patterns for genes encoding TonBs differed in interesting ways between DOM sources. Thus, whereas two TonB receptor genes were highly expressed in both DOMs, a TonB for assimilation of the *N*-acetylglucosamine (NAG) was specific to the exponential phase DOM and a TonB component involved in assimilation of iron was specific to the stationary phase DOM. The aminosugar NAG can be an abundant component of structural polysaccharides in phytoplankton like diatoms and dinoflagellates [41] and is the monomer that makes up chitin. The capacity to utilize NAG is widespread among marine bacteria (Riemann 2002) and pronounced changes in bacterial NAG uptake have been observed along the progression of a spring phytoplankton bloom (Eckert 2012). The differences in expression of alpha- and beta-glucosidases and an amylase further emphasize how different our studied bacteria reacted to DOM from distinct dinoflagellate growth phases. Regarding TonB receptors for iron, it is well established that they are involved in bacterial uptake of iron bound in siderophores, to supply iron that is an essential metal in enzymes for example in the respiratory chain. Interestingly, siderophore production is one of the mechanisms by which bacteria promote mutualistic relations with phytoplankton [42, 43], and possibly the expression of iron TonB receptors may reflect that iron is released to a higher degree by dinoflagellates in stationary compared to the exponential phase. In support of [5], our observed shifts in transcription suggest that changes in resources released across dinoflagellate growth phases (particularly polysaccharides) largely influence bacterial substrate utilization and growth.

### Comparison of gene expression between isolates

Beyond the transcriptional differences induced by exposure to DOM from different dinoflagellate growth phases within each of the studied bacteria, pronounced differences were noted between the two bacteria. This included differences already at the top level of the SEED classification system (e.g. in *Membrane transport* and *Fatty acids*), ant the unique expression in *Brevundimonas* BAL450 of a number of *Na+ H+ antiporters* (involved in pH and/or salinity adaptation [44]) and secretion system transporters (involved in adhesion, e.g. to algal cells [45]). Curiously, one of the most highly expressed genes in *Polaribacter* BAL334 was a transporter for phosphonate (also expressed in *Brevundimonas* BAL450 but at lower levels). Phosphonate is an organic form of phosphorus which can be used as a sole source of phosphorus by some microorganisms, allowing them higher fitness under phosphorus limiting conditions [46–48]. It is estimated that phosphonate constitutes a large fraction (5–25%) of the dissolved organic phosphorus (DOP) in the oceans [49]. During phosphate depletion in phytoplankton blooms, ABC-type phosphonate transporters proteins typically increase in abundance in some bacterial taxa [37]. Genes involved in phosphonate utilization are thus candidates to act as sensors for phosphate status in marine environments, complementing genes involved in phosphate utilization (e.g. phosphate membrane transporters and alkaline phosphatase) [50].

In the two model bacteria studied here, we found that the expression of *Ton and Tol transport systems* were dominant in both transcript abundance and in number of expressed genes. This class of transporters is found in the outer membrane of gram-negative bacteria and is involved in the uptake of a broad set of macromolecules, such as siderophores for iron, vitamin B_12_, nickel complexes and poly- or oligomeric carbohydrates [51]. Since DOM produced by phytoplankton can be rich in polysaccharides amenable to utilization by bacteria [52], expression of transporters for these types of compounds can be expected. It is particularly intriguing that we found so many different genes involved in the Ton and Tol systems expressed, as this is consistent with the uptake of not just a few preferred molecules, but the simultaneous uptake of a wide array of compounds exuded by phytoplankton. The characterization of sets of transporters in greater detail thus has the potential to provide deeper understanding of bacterial DOM metabolism along the progression of phytoplankton blooms, as recently shown from a coastal upwelling bloom where TonB transporters accounted for up to around 40% of the transcription of bacterial membrane transporters [53].

Interestingly, *Polaribacter* BAL334 also had higher relative expression of genes involved in the subsystems *Fatty acid metabolism cluster* and *Polyhydroxybutyrate metabolism*. PHB is produced by diverse bacteria in response to physiological stress or carbon excess [54]. The carbon stored in PHB can be used later as an energy source or as anabolic building blocks in times of low availability of DOM [54]. Interestingly, another flavobacterium, *Dokdonia* sp. MED134, has earlier been seen to express genes for a different carbon storage molecule–glycogen–under conditions where two strains of *Proteobacteria* expressed genes for PHB synthesis [55]. *Polaribacter* BAL334 encodes both pathways and the carbon storage strategy hence appears to not only reflect phylogenetic relatedness but also temporary ecological factors such as the composition of available substrates.

Even at the highest level of the SEED hierarchy, two categories stood out being exclusively expressed by just one of the two isolates: *Motility and Chemotaxis* in *Brevundimonas* BAL450 and *Photosynthesis* in *Polaribacter* BAL334. Genome analysis showed that *Polaribacter* BAL334 lacks the full complement of flagellar motility (it uses gliding motility for movement) [56]. In contrast, the flagellar motility system is present in *Brevundimonas* BAL450 where it was highly expressed. At the top level SEED, motility and chemotaxis gene expression was particularly high in the treatments with dinoflagellate DOM as compared to controls. This could potentially relate to the cells sensing increased nutrient availability in the DOM treatments or that the DOM provided energy that fueled increased swimming [57]. The *Photosynthesis* genes expressed by *Polaribacter* BAL334 were those encoding the energy-generating proteorhodopsin photosystem, which *Brevundimonas* BAL450 lacks. Strikingly, the two differentially abundant genes in proteorhodopsin synthesis were expressed at higher relative values in the controls than in the treatments with DOM. Proteorhodopsin is known to help bacterial cells to survive during starvation [58] or even contribute to growth at low DOC availability [59]. This suggests that *Polaribacter* BAL334 used the proteorhodopsin for surviving in the no-substrate controls, and when provided with DOM in the treated samples preferentially utilized DOM rather than photoheterotrophy for its energy demand.

## Conclusions

The relative expression responses we observed between bacterial species, and between DOM from two different dinoflagellate growth phases, helped identify genes potentially involved in shaping the ecology of heterotrophic marine microbes. Our findings emphasize the potential usefulness of experimental approaches for identifying indicator genes for different environmental conditions that are informative of mechanisms underlying important dynamics of carbon and nutrient fluxes in marine ecosystems. As such, our findings are encouraging for the future exploration of ecologically relevant patterns in experimental model systems and how different bacterial taxa respond to and/or transform DOM produced in relation to changes in phytoplankton’s physiological status across bloom development phases. Attaining sufficient precision in the identification of bacterial species in the field–for example through mapping of metatranscriptome data onto metagenomic assembled genomes (MAGs) [60, 61]–would allow the use of the genetic responses of particular species of marine bacteria beyond the confines of laboratory experiments to also interpret responses of bacteria as “living sensors” of the labile DOM spectrum encountered in their natural environment.

## Supporting information

S1 Table Expression of genes in the SEED category *Fatty Acids*, *Lipids*, *and Isoprenoids* in *Polaribacter* BAL334 and *Brevundimonas* BAL450. Both the SEED subcategory and subsystem are shown together with the expression abundance of the gene in counts per million (CPM) and standard deviation (CPM) in treatments and control. Note that a gene can occur in more than one SEED category.
(XLSX)

S2 Table Significantly differentially abundant genes in *Polaribacter* BAL334 and *Brevundimonas* BAL450 with SEED categories and SEED subsystems. Expressed genes were determined to be significantly more (denoted “up”) or less (denoted “down”) abundant in the transcriptomes of the DOM-enriched samples compared to controls, using EdgeR statistical analysis. Contrast indicates whether the gene occurs only in DOM from exponential phase (i.e. Exp-Con) or only in the stationary phase (i.e. Sta-Con) or is in both (i.e. shared genes). Note that a gene can occur in more than one SEED category.
(XLSX)
